# Endothelial Microparticles (EMP) for the Assessment of Endothelial Function: An In Vitro and In Vivo Study on Possible Interference of Plasma Lipids

**DOI:** 10.1371/journal.pone.0031496

**Published:** 2012-02-16

**Authors:** Sabrina H. van Ierssel, Vicky Y. Hoymans, Emeline M. Van Craenenbroeck, Viggo F. Van Tendeloo, Christiaan J. Vrints, Philippe G. Jorens, Viviane M. Conraads

**Affiliations:** 1 Department of Critical Care Medicine, Antwerp University Hospital (UZA), University of Antwerp (UA), Edegem, Belgium; 2 Laboratory of Cellular and Molecular Cardiology, Antwerp University Hospital (UZA), University of Antwerp (UA), Edegem, Belgium; 3 Department of Cardiology, Antwerp University Hospital (UZA), University of Antwerp (UA), Edegem, Belgium; 4 Centre for Cellular Therapy and Regenerative Medicine, Antwerp University Hospital (UZA), University of Antwerp (UA), Edegem, Belgium; Bristol Heart Institute, University of Bristol, United Kingdom

## Abstract

**Background:**

Circulating endothelial microparticles (EMP) reflect the condition of the endothelium and are of increasing interest in cardiovascular and inflammatory diseases. Recently, increased numbers of EMP following oral fat intake, possibly due to acute endothelial injury, have been reported. On the other hand, the direct interference of lipids with the detection of EMP has been suggested. This study aimed to investigate the effect of lipid-rich solutions, commonly administered in clinical practice, on the detection, both in vitro and in vivo, of EMP.

**Methods:**

For the in vitro assessment, several lipid-rich solutions were added to whole blood of healthy subjects (n = 8) and patients with coronary heart disease (n = 5). EMP (CD31+/CD42b−) were detected in platelet poor plasma by flow cytometry. For the in vivo study, healthy volunteers were evaluated on 3 different study-days: baseline evaluation, following lipid infusion and after a NaCl infusion. EMP quantification, lipid measurements and peripheral arterial tonometry were performed on each day.

**Results:**

Both in vitro addition and in vivo administration of lipids significantly decreased EMP (from 198.6 to 53.0 and from 272.6 to 90.6/µl PPP, respectively, p = 0.001 and p = 0.012). The EMP number correlated inversely with the concentration of triglycerides, both in vitro and in vivo (r = −0.707 and −0.589, p<0.001 and p = 0.021, respectively). The validity of EMP as a marker of endothelial function is supported by their inverse relationship with the reactive hyperemia index (r = −0.758, p = 0.011). This inverse relation was confounded by the intravenous administration of lipids.

**Conclusion:**

The confounding effect of high circulating levels of lipids, commonly found in patients that receive intravenous lipid-based solutions, should be taken into account when flow cytometry is used to quantify EMP.

## Introduction

Circulating endothelial microparticles (EMP) are increasingly studied as markers of endothelial function in cardiovascular and inflammatory diseases [1]. EMP are shed into the peripheral circulation upon endothelial cell activation, apoptosis and injury, and are therefore suited to reflect endothelial damage. Besides being markers of endothelial function, EMP may have important functional properties [2]. As such their modulating role in inflammation, vascular function and coagulation is increasingly recognized [2]. Flow cytometry is most often applied to detect and quantify EMP [3]. Since EMP precipitation may cause loss of microparticles (MP), measurements are mainly performed directly in plasma [4].

In many critically ill patients, dysfunction of the endothelium is an important factor, contributing to disease severity and progression. Hence, there is growing interest in the evaluation of the endothelial function in these patients using both in vivo measurements and circulating markers, including microparticles [5], [6], [7]. This group of patients also frequently receives lipid solutions, either as a component of total parenteral nutrition (TPN) or as part of the unique formulation of the sedative agent propofol, resulting in a high prevalence of hyperlipidemia [8], [9].

Lipemic plasma is well known to interfere with various laboratory techniques, especially with those methods that use light transmission as a detection manner. With regard to flow cytometry lipemia, and particularly chylomicrons, impede platelet detection [10]. In addition, several recent reports have shown higher numbers of circulating EMP after a high fat meal, a finding that was interpreted to reflect acute endothelial dysfunction [11], [12], [13]. However, the direct influence of lipids on the flow cytometric detection of EMP has not yet been investigated.

The aim of the present study was to assess the effect of a lipid-rich solution on EMP quantification by flow cytometry, both in vitro and in vivo. First, a pure lipid solution, TPN or propofol (the last two also contain high amounts of lipids) were added in vitro to whole blood from healthy subjects and patients with coronary heart disease. Second, we administered a parenteral lipid emulsion to healthy individuals and determined the effects on the numbers of EMP and in vivo endothelial function.

## Results

### In vitro study

**Figure 1:**
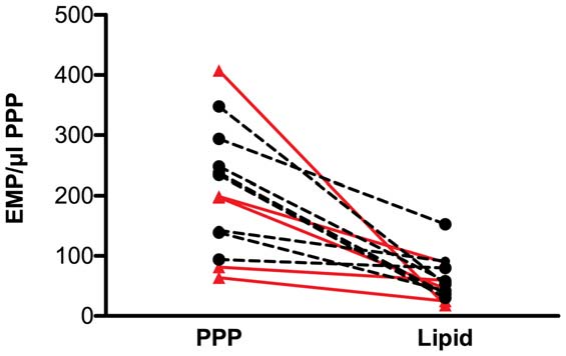
Decrease in EMP after in vitro administration of lipid-rich solutions. Whole blood was taken from 8 healthy volunteers (black) and 5 patients with coronary heart disease (red). PPP was prepared from different aliquots to which lipid-rich solutions were added in different concentrations. EMP were detected by flow cytometry as particles <1 µm and CD31+/CD42b−. The figure shows EMP numbers/µl PPP from samples without lipid-rich solutions (PPP), and samples with added lipid-rich solutions in the lowest concentration (Lipid). The number of EMP detected by flow cytometry decreased significantly (all data taken together p = 0.001). EMP = endothelial microparticles, PPP = platelet poor plasma.

**Figure 2:**
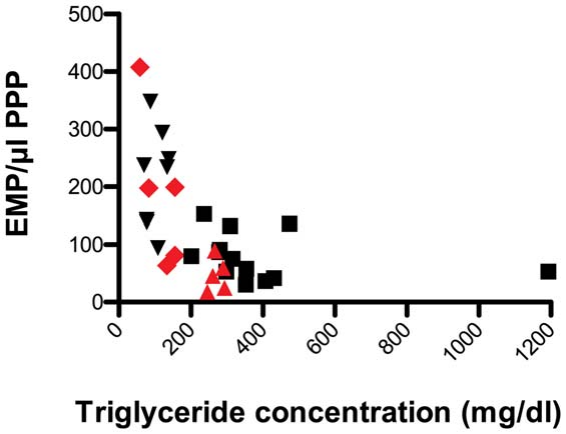
The inverse relation between EMP and TG after the in vitro administration of lipid-rich solutions to whole blood. Whole blood was taken from 8 healthy volunteers (black) and 5 patients with coronary heart disease (red). PPP was prepared from different aliquots to which lipid-rich solutions were added in different concentrations. EMP were detected by flow cytometry as particles <1 µm and CD31+/CD42b−, and TG concentration were determined in plasma. The figure shows EMP numbers/µl PPP from samples without lipid-rich solutions (black inversed triangles for the healthy volunteers and red diamonds for the cardiovascular patients) and samples with added lipid-rich solutions in different concentrations (black quadrangles for the healthy volunteers and red triangles for the cardiovascular patients). Taken all samples together a non-linear inverse relation exists between TG concentration and the number of EMP (r = −0.707, p<0.001). EMP = endothelial microparticles, PPP = platelet poor plasma, TG = triglycerides.

Results are shown in table 1. All healthy volunteers (n = 8) had baseline fasting triglyceride (TG) concentrations below 150 mg/dl. In the group of cardiovascular patients (n = 5) the triglyceride concentration was 133.3 mg/dl (58.5–155.8). In both study-populations separately and grouped the addition of lipid-rich solutions resulted in a significant increase of TG concentration (p<0.05, in all 3 situations). A lower number of EMP was detected by flow cytometry after the addition of lipids in all study subjects (Figure 1). This decrease was significant in the two groups apart and taken together (p<0.05, in all 3 situations) (Table 1). A non-linear relation exists between the number of EMP and the TG concentration in plasma (taken all in vitro data together, r = −0.707, p<0.001) (Figure 2).

**Table 1:** In vitro study results.

	Healthy controls (N = 8)	Cardiovascular patients (N = 5)	Total (N = 13)
**TG in PPP (mg/dl)**	98.0 (69.9–138.4)	133.3 (58.5–155.8)	108.4 (58.5–155.8)
**TG in lipid sample (mg/dl)** *	325.5 (201.4–617.7)$	225.7 (208.5–248.9)§	289.4 (201.4–617.7)**
**EMP in PPP (/µl PPP)**	253.4 (93.9–347.6)	197.2 (63.6–407.3)	198.6 (63.6–407.3)
**EMP in lipid sample (/µl PPP)** *	55.4 (30.1–152.5)$	45.2 (17.9–88.9)§	53.0 (17.9–152.5)**

*For every volunteer/patient the sample with the lowest amount of lipid-rich solutions added was used.

$ Compared to PPP Wilcoxon signed rank test, p = 0.012.

§ Compared to PPP Wilcoxon signed rank test, p = 0.043.

**Compared to PPP Wilcoxon signed rank test, p = 0.001.

Whole blood was taken from 8 healthy volunteers and 5 patients with coronary heart disease. PPP was prepared from different aliquots to which lipid-rich solutions were added in different concentrations. EMP were detected by flow cytometry as particles <1 µm and CD31+/CD42b−, and TG concentration were determined in plasma. EMP = endothelial microparticles, TG = triglycerides, PPP = platelet poor plasma.

### In vivo study

**Figure 3:**
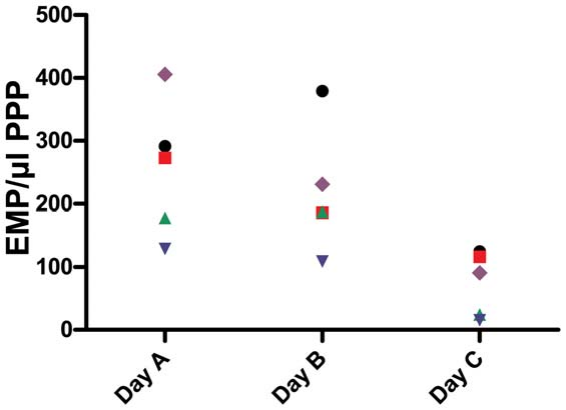
Evolution of EMP detected by flow cytometry in the in vivo study. 5 healthy volunteers were evaluated on 3 different study days (all 5 represented in a different color and symbol). Day A: blood was collected after an overnight fast. EMP were detected by flow cytometry as particles <1 µm and CD31+/CD42b− in PPP. Day B: NaCl 0.9% was administered in fasting conditions and the same measurements as on day A were performed. Day C: the same protocol was used as on day B, but a pure lipid solution was infused instead of NaCl. A lower number of EMP was detected in all healthy volunteers after intravenous administration of the lipid emulsion (p = 0.012). EMP = endothelial microparticles, PPP = platelet poor plasma.

Results are shown in Table 2. The in vivo infusion of a pure lipid solution caused an increase in both plasma TG concentration and chylomicron fraction (p = 0.015 and 0.018, respectively). There was also a trend towards a higher very low-density lipoprotein (VLDL) fraction after lipid infusion, although not statistically significant (p = 0.074). No differences were found in the total cholesterol concentration, high-density lipoprotein (HDL) fraction or low-density lipoprotein (LDL) fraction. A lower number of EMP was detected in all healthy volunteers after intravenous administration of the lipid emulsion (day C, p = 0.012), i.e. in accordance with the in vitro data (Figure 3). There was no statistically significant difference in reactive hyperemia index (RHI) when measured on the various study-days.

**Table 2:** In vivo study: results for the different study-days.

	Day A	Day B	Day C
**TG (mg/dl)**	87.5 (70.0–131.5)	68.9 (59.5–118.1)	202.2 (149.5–278.9)*
**CH (mg/dl)**	184.5 (170.3–188.5)	190.0 (176.9–198.9)	185.2 (169.2–192.1)
**LDL (%)**	53.6 (46.8–61.9)	51.1 (46.7–61.7)	48.5 (43.4–58.5)
**HDL (%)**	42.0 (31.6–44.2)	43.6 (31.6–46.5)	36.2 (26.3–46.2)
**VLDL (%)**	7.0 (4.5–9.1)	5.0 (4.6–6.2)	9.1 (5.4–11.0)
**Chyl (%)**	1.3 (1.2–1.4)	0.0 (0.0–2.2)	4.3 (4.1–7.5)*
**EMP/µl PPP**	272.6 (128.6–405.6)	187.3 (108.6–379.1)	90.6 (15.2–124.6)*
**RHI**	1.60 (1.46–2.1)	1.90 (1.07–2.65)	1.38 (1.34–2.55)

Results are expressed as median (min-max).

*Friedman test p<0.05 and Wilcoxon signed rank test Day C vs. Day B and Day C vs. Day A p<0.05.

5 healthy volunteers were evaluated on 3 different study days. Day A: blood was collected after an overnight fast and PAT was performed. EMP were detected by flow cytometry as particles <1 µm and CD31+/CD42b− in PPP. Day B: a NaCl 0.9% infusion was administered in fasting conditions and the same measurements as on day A were performed. Day C: the same protocol was used as on day B, but a pure lipid solution was infused instead of NaCl.

CH = cholesterol, chyl = chylomicrons, EMP = endothelial microparticles, HDL = High density lipoproteins, LDL = low density lipoproteins, PAT = peripheral arterial tonometry, RHI = reactive hyperemia index, TG = triglycerides, VLDL = very low density lipoproteins.

**Figure 4:**
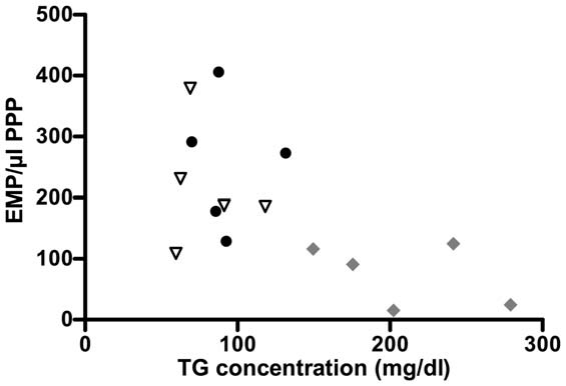
In vivo there is a non-linear inverse relation between TG concentration and EMP detected in plasma. 5 healthy volunteers were evaluated on 3 different study days. Day A: blood was collected after an overnight fast. EMP were detected by flow cytometry as particles <1 µm and CD31+/CD42b− in PPP, and lipid profile was determined on frozen plasma samples. (Black dots) Day B: NaCl 0.9% was administered in fasting conditions and the same measurements as on day A were performed. (Black open triangles) Day C: the same protocol was used as on day B, but a pure lipid solution was infused instead of NaCl. (Gray diamonds) There is an inverse non-linear relation between the TG concentration in plasma and the number of EMP number/µl PPP detected (r = −0.589, p = 0.021). EMP = endothelial microparticles, PPP = platelet poor plasma, TG = triglycerides.

The in vivo study showed an inverse relation between the TG concentration and EMP numbers (r = −0.589, p = 0.021), as well (Figure 4). A similar inverse relation was found between EMP numbers, the chylomicron fraction and the VLDL fraction (r = −0.700 and r = −0.545, p = 0.004 and 0.036, respectively).

**Figure 5:**
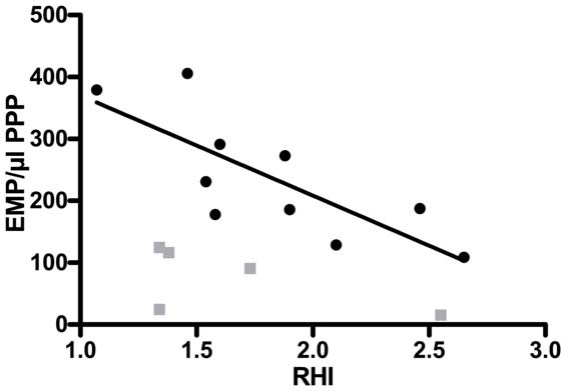
In healthy volunteers the inverse relation of EMP with RHI is confounded by the intravenous administration of a pure lipid solution. 5 healthy volunteers were evaluated on 3 different study days. Day A: blood was collected after an overnight fast and PAT was performed. EMP were detected by flow cytometry as particles <1 µm and CD31+/CD42b− in PPP. Day B: a NaCl 0.9% infusion was administered in fasting conditions and the same measurements as on day A were performed. Day C: the same protocol was used as on day B, but a parenteral lipid emulsion was infused instead of NaCl. On day A and B (black dots) there was an inverse relation between EMP numbers/µl PPP and RHI (r = −0.758, p = 0.011 combining data from day A and day B). This relation was confounded by lipid infusion as on day C (gray quadrangles) (r = −0.254, p = 0.361 combining all data). EMP = endothelial microparticles, PAT = peripheral arterial tonometry, PPP = platelet poor plasma, RHI = reactive hyperemia index.

Combined results obtained on day A and B, showed an inverse linear relation between EMP and RHI (r = −0.758, p = 0.011) (Figure 5). This inverse relationship was confounded when all data were taken into account, i.e. including those after intravenous lipid infusion (r = −0.254, p = 0.361 combining all data) (Figure 5).

## Discussion

We investigated the effects of lipid-rich solutions on the flow cytometric detection of plasma EMP, both in vitro and in vivo. Several findings emerged from this study. First, both in vitro and in vivo administration of lipid-rich solutions lowered the number of EMP detected by flow cytometry in healthy volunteers. The in vitro effect was further confirmed in a group of 5 patients with known cardiovascular disease. Second, EMP numbers related inversely to plasma TG concentrations. Last, there was a negative correlation between baseline EMP numbers and RHI, obtained with peripheral arterial tonometry (PAT). This finding underscores the value of EMP as markers of endothelial function.

The endothelium plays a key role in the regulation of vascular tone, barrier function, coagulation, inflammation and angiogenesis [14]. Circulating factors, such as EMP, in addition to the in vivo assessment of endothelial function, are increasingly used to evaluate endothelial damage. EMP express membrane proteins, which are related to their endothelial origin, but also depend on the process that causes their release [15]. For example, apoptosis of endothelial cells increases the numbers of circulating CD31+/CD42b− EMP. Besides the fact that EMP reflect the “general condition” of the endothelium, there is a growing awareness that these particles may act as small vectors, transporting proteins, lipids, DNA, RNA and miRNA, through which they modulate inflammation, vascular function and coagulation [2].

### Lipids and endothelial function

The effect of lipids on EMP measurements was mostly studied after the administration of a lipid-rich meal [11], [12], [13], [16]. Generally, a higher circulating number of postprandial EMP was found in these studies. These data have been interpreted as the result of postprandial endothelial damage, involving hypertriglyceridemia, hyperglycemia and hyperinsulinemia [17], [18]. In most studies TG concentrations obtained after a lipid-rich meal, were lower than the values achieved in the present study. As illustrated in figure 2 and 4, the non-linear relation between EMP and TG suggests that there might be a certain threshold, above which interference with EMP quantification becomes significant after the addition of lipid-rich solutions.

The lipid-rich solutions that were used in the present study, obviously differ from the physiological nutritional and hormonal situation obtained after a high fat meal [19]. The lipid composition of intralipid is fixed (1.2% phospholipids, 20% TG containing predominantly unsaturated fatty acids and 2.25% glycerin), while the lipid composition (saturated, unsaturated fatty acids and cholesterol) of high fat meals differs between studies. We intravenously administered low doses of fat (12 g or 6 g/h) in the form of chylomicron-like particles, without the administration of carbohydrates or proteins. As such there are quantitative and qualitative differences with regard to chylomicrons and chylomicron-like lipid particles post-intravenous lipid administration, compared to the situation after a lipid-rich meal. Although we cannot exclude that the latter may also have affected endothelial function, further elucidation of this possible interaction was beyond the scope of our study.

The possible influence of an intravenously administered pure lipid solution on the detection of EMP has never been reported until now. With regard to the effect of exogenous lipid administration on in vivo endothelium-dependent vasodilation, conflicting results have been described [20], [21]. In the present study we found a decreased number of CD31+/CD42b− EMP, and no difference in RHI.

### Lipids and flow cytometry for EMP

A significant number of pre-analytical and analytical variables has been described, all of which appear to confound exact EMP quantification [22]. Within the International Society of Thrombosis and Haemostasis, great effort has been put into standardization of the pre-analytical and analytical issues, but a widely adopted protocol is still lacking. Storage time of whole blood and plasma, and centrifugation speed are known to influence MP measurement, all of which were controlled in this particular study. Since microparticles are measured mostly directly in PPP, it is possible that other components present in plasma, such as lipids, interfere with EMP detection. Several reports describe elevated EMP numbers after a high fat meal, a phenomenon that is considered to reflect acute endothelial dysfunction [17], [18]. However, data on the possible interference of lipids with the flow cytometric quantification of EMP are scarce.

In lipemic plasma, chylomicrons and VLDL cause light scattering and lead to turbidity and cloudiness [19]. The size of microparticles is in the range of chylomicrons, VLDL and artificial lipid particles. Previous studies showed that chylomicrons appear in the forward (FSC) and side scatter (SSC) region of interest for microparticle analysis, and can interfere with MP detection [23]. Chylomicrons, VLDL and artificial lipid particles are present in plasma during the administration of a pure lipid solution, and might therefore at least partly explain the current findings. Besides interference by light scattering; antigen-antibody-binding and interactions in the pre-analytical methods, e.g. upon centrifugation, can also occur. Further investigation on the mechanisms underlying this interference is advocated.

To avoid interference with microparticle analyses in lipid-rich plasma samples, Shet et al suggested that the upper lipid layer is to be avoided during pipetting [3]. In our hands the lipids are not always clearly delineated, so avoiding them can be difficult. Another possible solution is the isolation of MP from plasma by high speed centrifugation, as applied by Sustar et al [16]. Since lipids shift upwards in plasma during centrifugation, it is likely that the majority will be removed with the supernatants, thereby avoiding interference with flow cytometric detection [24]. High-speed centrifugation (13 000 g for 45 minutes) of plasma diluted with buffer allows the removal of the plasma containing lipids. Nevertheless, not all plasma microparticles will be sedimented by high speed centrifugation [4]. Despite diluting plasma in buffer to decrease the density, we could only spin down around two third of the EMP (own data not shown). Furthermore, since the sedimentation of microparticles depends on the density of the plasma, we do not exactly know what the influence of the additional lipids will be on the sedimentation rate.

The limited number of volunteers evaluated in the in vivo part of this study, could explain the lack of a statistically significant difference in terms of endothelial function following the administration of a pure lipid solution. However, our results clearly show that the inverse relation between EMP numbers and endothelial function is confounded after lipid infusion. (Figure 5) This observation suggests that lower EMP numbers are a technical artifact attributable to interference of lipids. Furthermore, in vitro and in vivo data point into the same direction, and show a uniform lipid-induced reduction in EMP numbers in all volunteers. (Figure 1 and 3)

Our findings may have practical implications for future research. Besides the determination of EMP as a surrogate marker for endothelial function in cardiovascular diseases, it is also increasingly used in critically ill [2], [5]. The latter group is frequently exposed to intravenous lipid administration, as most sedated patients receive high amounts of propofol formulated as a lipid emulsion. Furthermore, many sick patients both in and outside the ICU also receive TPN, a solution rich in lipids. Both treatments lead to a significant elevation of TG levels, which are well in the range of those observed in our healthy volunteers [8], [9]. In those patients in particular, the interference of high lipid levels on EMP numbers should be taken into account.

### Conclusion

While the relation between EMP, as a marker of endothelial function, and lipidemia is an important topic in the current literature, little is known about the possible interference of lipemic plasma on the flow cytometric detection of EMP. We observed that, both in vitro and in vivo, lipid-rich solutions lower the numbers of EMP detected by flow cytometry in healthy volunteers and cardiovascular patients. This decrease is inversely and non-linearly related to the concentration of TG in plasma. The inverse relationship between EMP and RHI, underscores the valid use of EMP as a marker of endothelial function. However, this inverse relation is disturbed by the presence of high lipid levels. This strongly suggests that in lipemic samples, the interpretation of EMP measurements by flow cytometry needs to be executed with caution.

## Materials and Methods

The study protocol was approved by the research and ethics committee of the Antwerp University Hospital, corresponding to the principles outlined in the Declaration of Helsinki (B30020108323). A written informed consent was obtained from all subjects.

### In vitro study

**Figure 6:**
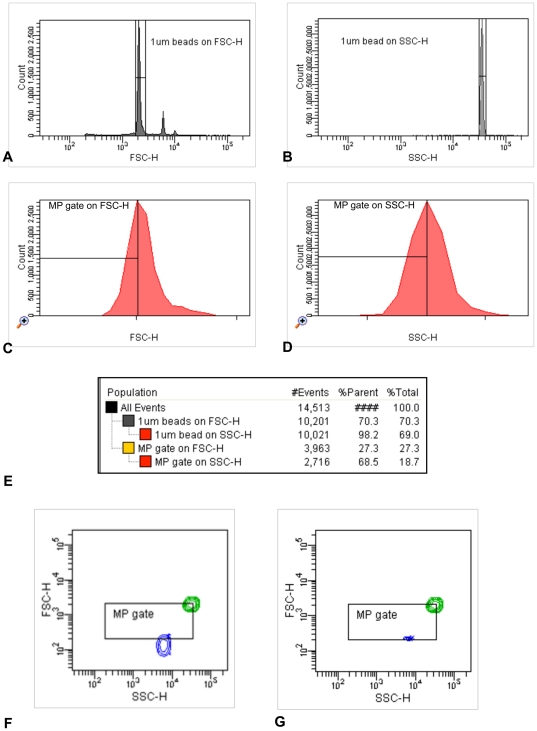
Gating strategy microparticle dimension criteria. Fluoresbrite YG 1 µm calibration size beads (Polysciences, Eppelheim Germany) were used to set forward (FSC) and side scatter (SSC) criteria for the assessment of microparticles. Beads were measured at low flow rate and threshold was placed on both FSC and SSC. More in particular, the MP analysis region was defined as follows: at first, the bead population was selected on a histogram of the FSC signal (A), while the events within this gate were further selected on SSC signal (B); the upper detection limit of the microparticle gate was set on the peak of the selected 1 µm beads on histogram plots of FSC (C) and SSC (D). For illustrative purposes, the population hierarchy is shown in figure E. The lower dimension criterion was set just above the electronic noise of the cytometer, which corresponded with the upper boarder of the Fluoresbrite YG 0.5 µm calibration size beads on FSC as illustrated in figures F and G. In figure F, the threshold was placed on FITC, and in figure G, the threshold was placed on FSC and SSC. As an illustration our microparticle dimension criteria are shown as a rectangular gate in figure F and G. The position of the peak signal of the 1 µm beads on FSC and SSC was maintained at the same place by adjusting the FSC and SSC voltage when necessary. FITC = fluorescein, FSC-H = forwards scatter height, SSC-H = sight scatter height, MP = microparticles, PPP = platelet poor plasma.

**Figure 7:**
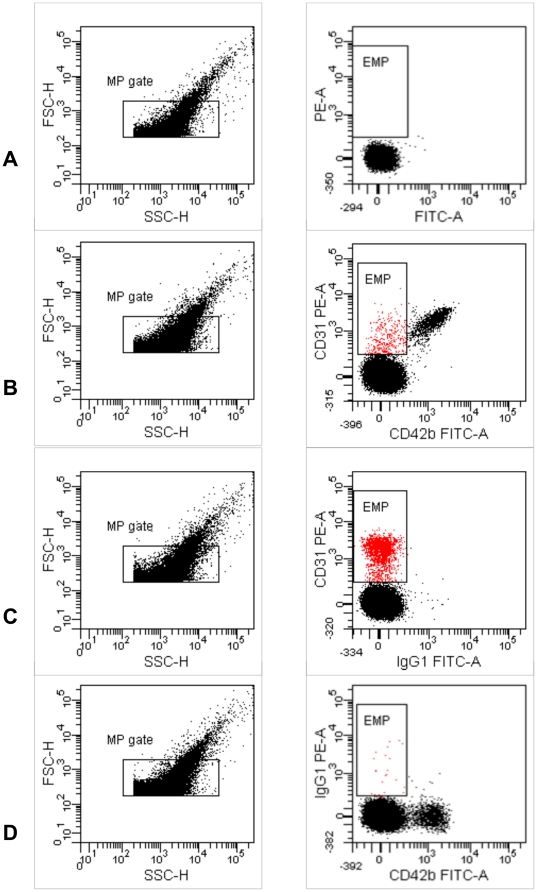
Microparticle gating strategy. For the detection of EMP, PPP was incubated at 4°C for 20 minutes with CD31-PE (BD Biosciences, Erembodegem Belgium) and CD42b-FITC (BD Bioscience, Erembodegem Belgium), both 0.22 µm filtered and azide free. Samples were diluted in 0.22 µm filtered FACSflow (BD Biosciences, Erembodegem Belgium), to allow sample acquisition at less than 1000 events/sec on low flow rate. Samples were analyzed on a FACSCantoII (BD Biosciences, Erembodegem Belgium). Antibodies were titrated, and isotype fluorescence-minus-one for FITC (C) and PE (D) and unstained samples (A) were run as controls. EMP were defined as particles smaller than 1 µm that were CD31-positive and CD42b-negative (B). For illustrative reasons the MP gate is shown as a rectangular gate. For the settings of the dimension criteria see figure 6. EMP = endothelial microparticle, FITC = fluorescein, FSC-H = forwards scatter height, SSC-H = sight scatter height, MP = microparticle, PE = phycoerythrin, PPP = platelet poor plasma.

Blood was sampled from 8 healthy volunteers (median [min-max] age 30 [26–49] years, 7 females, no cardiovascular risk factors), after an overnight fast. Furthermore 5 patients with known coronary heart disease were included (characteristics in Table 3). One had coronary vasospasms and the 4 others had coronary atheromatosis. Blood sampling procedure was performed using ACD vacutainer tubes (BD diagnostics, Erembodegem Belgium) as previously described [22]. Lipid-rich solutions were added to different aliquots of whole blood: propofol 1% (Diprivan®, AstraZeneca) 1.2 µl/ml, 2.4 µl/ml or 4.8 µl/ml (i.e. 0.12, 0.24 and 0.48 mg lipids added/ml whole blood, respectively) (n = 2), TPN containing SMOFlipid® (Fresenius Kabi) 35 ul/ml and 70 µl/ml (n = 1) (i.e. 1.16 and 2.33 mg lipids added/ml blood, respectively), a pure lipid solution (Intralipid® 20%, Fresenius Kabi) 2.5 µl/ml (i.e. 0.5 mg lipids added/ml whole blood) (n = 4) or 5 µl/ml (i.e. 1 mg lipids added/ml whole blood) (n = 1). The amount of lipid-rich solutions added to the plasma was chosen to achieve conditions comparable to those obtained after the in vivo administration of TPN or propofol in patients. In short, all samples were mixed gently at room temperature for 15 minutes, then PPP was prepared by two 20 minute centrifugations at 1550 g without acceleration or break. The samples with and without the addition of lipid-rich solutions were all processed in the same manner. Since the amount of circulating EMP in the plasma of healthy volunteers is low, we chose the most robust marker for EMP detection, i.e. CD31+/CD42b− [1], [22]. For the detection of EMP, PPP was incubated at 4°C for 20 minutes with CD31-PE (BD Biosciences, Erembodegem Belgium) and CD42b-FITC (BD Bioscience, Erembodegem Belgium), both 0.22 µm filtered and azide free. Samples were diluted in 0.22 µm filtered FACSflow (BD Biosciences, Erembodegem Belgium), to allow sample acquisition at less than 1000 events/sec on low flow rate. Samples were analyzed on a FACSCantoII (BD Biosciences, Erembodegem Belgium). The upper detection limit of our microparticle gate on FSC and SSC was established using Fluoresbrite YG 1 µm calibration size beads (Polysciences, Eppelheim Germany). (Figure 6) The lower detection limit, a FSC and SSC threshold, was placed above the electronic noise of our cytometer; this corresponds with the upper boarder of 0.5 µm beads on FSC. (Figure 6) The position of the peak signal of the 1 µm beads on FSC and SSC was maintained at the same place. Background noise in 0.22 µm filtered FACS flow was checked daily so that it was less than 100 events/sec. Trucount tubes (BD Biosciences, Erembodegem Belgium) were run in parallel to determine final MP concentration per µl PPP. EMP were defined as particles smaller than 1 µm that were CD31-positive and CD42b-negative [22].(Figure 7) Antibodies were titrated, and isotype fluorescence-minus-one and unstained samples were run as controls. All samples were measured in duplicate. Mean coefficient of variation was 6.84% for PPP and 10.11% for lipid-rich PPP. Antibody binding specificity was verified in lipid-poor and lipid-rich samples by a competition assay using excess (10 times) of unconjugated CD31 and CD42b antibody (BD Biosciences, Erembodegem Belgium), after titration (Data not shown). The pure lipid emulsion in buffer (0.22 µm filtered FACSflow) caused a typical scatter pattern on FSC-SSC, which we could retrieve in our lipid-rich plasma samples, and was not autofluorescent (Data not shown).

**Table 3:** Characteristics of cardiovascular patients (n = 5).

**Age years (median [min-max])**	59 (50–65)	**Coronarography results**	
**Gender (F/M)**	1/4	*Significant stenosi*s	3
**Body mass index median [min-max])**	24.0 (22.7–31.6)	Non significant stenosis	1
**Cholesterol mg/dl (median [min-max])**	137 (117–211)	**Treatment**	
**Cardiovascular (CV) risk factors**		*ACE-inhibitor or sartan*	4
*-Familial history of CV disease*	3	*β-Blocker*	2
*-Smoking (active)*	3 (1)	*Acetylsalicylic acid*	5
*-Arterial hypertension*	4	*Statin*	3
*-Hypercholesterolemia*	4		
*-Diabetes (insulin dependent)*	2 (1)		

CV disease = cardiovascular disease.

All plasma samples, both PPP without and with added lipid-rich solutions, were analyzed for triglyceride (TG) concentration using Vitros Ortho Clinical Diagnostics (Johnson & Johnson).

### In vivo study

5 healthy volunteers (median [min-max] age 27 [26–30] years; 3 females; no cardiovascular risk factors) were evaluated on 3 different study-days, applied in random order. On day A, blood was collected after an overnight fast. PPP was prepared immediately for EMP detection and plasma samples were frozen at −80°C for lipid measurements. In vivo assessment of endothelial function was performed (see further) after 30 minutes. On day B, fasting volunteers received a NaCl 0.9% infusion (0.5 or 1 ml/min) during 1 hour, after which the same laboratory and clinical measurements were performed as on day A. The infusion continued during these measurements. Day C was similar to day B, but a parenteral pure lipid emulsion (Intralipid® 20%, AstraZeneca) (0.5 or 1 ml/min) was infused instead of NaCl 0.9%. Two different infusion speeds were chosen to obtain different triglyceride concentrations.

For the infusion, a peripheral catheter was placed in the dominant arm. Blood was collected from the non-dominant arm. EMP were detected in fresh PPP using flow cytometry as described above. All samples were run in duplicate. Mean coefficient of variation for Day A, B and C was 4.21, 7.38 and 11.37%, respectively.

The lipid profile was determined on plasma samples frozen at −80°C using Dimension Vista 1500 Systems (Siemens). The lipoprotein composition was evaluated using zone electrophoresis on agarose gels (Hydragel Lipo, Sebia), and expressed as a percentage of the total lipoprotein content.

For the in vivo assessment of endothelial function, peripheral arterial tonometry (PAT), endoPAT® (Itamar, Israel) was used in accordance to the guidelines published by the European Society of Cardiology [25]. Subjects were analyzed in a quiet room after acclimatization, and at least 30 minutes after the blood collection. Suprasystolic occlusion of the non-dominant upper arm, at 200 mmHg or 60 mmHg above systolic blood pressure, was obtained using a cuff. The reactive hyperemia index (RHI) was calculated with the Itamar software (version 3.2.4).

### Statistics

The statistical program SPSS version 18 was used for data analysis. Due to the limited sample size, non-parametric tests were used. Data are expressed as median (min-max). Wilcoxon signed rank test was used to compare groups in the in vitro study. To compare the 3 different study-days in the in vivo study, the Friedman test was applied and if significant, a Wilcoxon signed rank test was performed comparing the different study-days two by two. Spearman correlation coefficient was used to evaluate the relation between TG lipid profile, EMP number and PAT measurements.
